# The carbon chain-selective adenylation enzyme TamA: the missing link between fatty acid and pyrrole natural product biosynthesis†
†Electronic supplementary information (ESI) available: Experimental section and results section containing sequence alignments, protein purification, mass spectra and structural models. See DOI: 10.1039/c8ob00441b


**DOI:** 10.1039/c8ob00441b

**Published:** 2018-03-22

**Authors:** Piera M. Marchetti, Van Kelly, Joanna P. Simpson, Mairi Ward, Dominic J. Campopiano

**Affiliations:** a EaStCHEM School of Chemistry , David Brewster Road , University of Edinburgh , Edinburgh , EH9 3FJ , UK . Email: Dominic.Campopiano@ed.ac.uk

## Abstract

The marine bacterium *Pseudoalteromonas tunicata* produces the bipyrrole antibiotic tambjamine YP1. This natural product is built from common amino acid and fatty acid building blocks in a biosynthetic pathway that is encoded in the *tam* operon which contains 19 genes. The exact role that each of these Tam proteins plays in tambjamine biosynthesis is not known. Here, we provide evidence that TamA initiates the synthesis and controls the chain length of the essential tambjamine fatty amine tail. Sequence analysis suggests the unusual TamA is comprised of an N-terminal adenylation (ANL) domain fused to a C-terminal acyl carrier protein (ACP). Mass spectrometry analysis of recombinant TamA revealed the surprising presence of bound C11 and C12 acyl-adenylate intermediates. Acylation of the ACP domain was observed upon attachment of the phosphopantetheine (4′-PP) arm to the ACP. We also show that TamA can transfer fatty acids ranging in chain length from C6–C13 to an isolated ACP domain. Thus TamA bridges the gap between primary and secondary metabolism by linking fatty acid and pyrrole biosynthetic pathways.

## Abstract 2

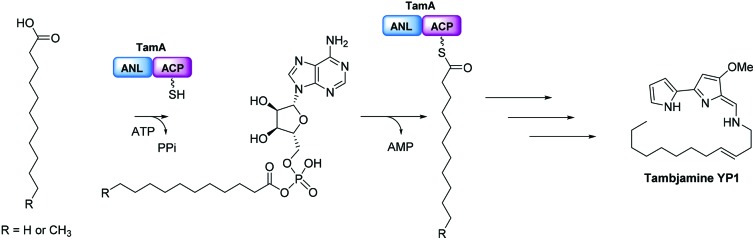
TamA is the adenylating enzyme that selects and activates fatty acids for tambjamine biosynthesis.

## Introduction

**Fig. 1:**
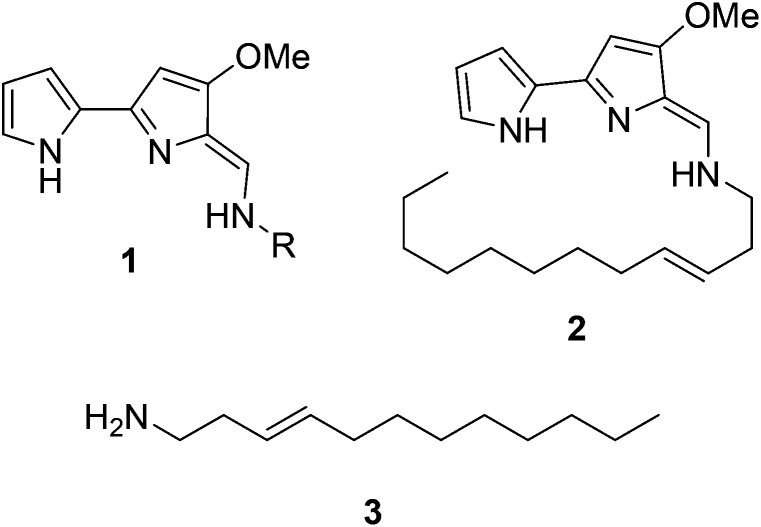
General structure of the bipyrrole core of the tambjamines (**1**, R represents acyl chains ranging from C2 to C12), structure of tambjamine YP1 (**2**) and structure of the proposed amine intermediate that forms the tambjamine YP1 tail (**3**).

Natural products have long been excellent sources of drugs with natural product based treatments comprising over 50% of small molecules approved for medical use in the last 35 years.^1^ The tambjamines (**1**, Fig. 1) are a family of yellow natural products isolated from various marine organisms^2^,^3^ that display antimicrobial,^2^,^4^ antimalarial^5^ and mammalian cytotoxic activity.^4^,^6^–^8^ They contain a core bipyrrole structure that allows anion coordination and transport^9^,^10^ as well as DNA intercalation.^11^ In addition, their essential hydrocarbon tail is thought to facilitate their crossing of cell membranes.^12^ These properties suggest that tambjamines have promising, broad, clinical potential against various diseases including cystic fibrosis, where natural ion transporters are impaired.^9^

To date, the most hydrophobic natural tambjamine that has been characterised is tambjamine YP1^13^ (**2**, Fig. 1). It is also the only tambjamine whose biosynthetic gene cluster from *Pseudoalteromonas tunicata* has been identified^14^ (Fig. S1†). The YP1 cluster is proposed to contain 19 open reading frames (ORFs) that encode enzymes which assemble the bipyrrole product from amino acid and fatty acid building blocks (Fig. S2, Table S1†). The majority of these enzymes have been assigned putative functions based on the biosynthesis of various natural products, including a similar class of molecules, the tri-pyrrole containing prodiginines.^15^,^16^ Biosynthesis of the YP1-specific tail was thought to culminate in a long chain amine (**3**, Fig. 1) which attaches to the bipyrrole ring system by an enamine linkage. However, the exact details of how this functionality is produced has not been explored.

Kjelleberg and colleagues postulated that the amine tail is derived from C12 lauric acid.^14^ In *P. tunicata* this is likely to be produced by the fatty acid synthase (FAS) machinery.^17^,^18^ The YP1 biosynthetic pathway then harnesses the FAS output and converts it to a long chain amine. This can be achieved by the commonly used mechanism of fatty acid activation, namely; the adenylation of the acid using ATP, followed by reaction with coenzyme A (CoASH) to generate a thioester intermediate (Fig. S3†). Previously it was predicted that this transformation is carried out by AfaA, a fatty acid CoA ligase (FACL) from outside the Tam cluster. The resulting C12–CoA thioester intermediate would then be subjected to downstream processing by Tam cluster enzymes to eventually yield the amine (**3**, Fig. 1).

## Results and discussion

**Fig. 2:**
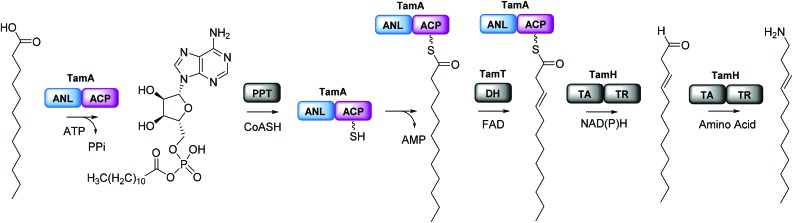
Proposed pathway for the formation of the tambjamine YP1 C12 amine tail in *P. tunicata*. This begins with acyl-adenylate formation catalyzed by the N-terminal TamA adenylation domain (ANL, blue) and transfer of the fatty acid to the acyl carrier protein (ACP) domain (purple) which has been converted to the phosphopantetheine (4′-PP) form by a 4′-PP transferase (PPT) using coenzyme A (CoASH). The TamA-bound acyl-thioester is then dehydrogenated by TamT (dehydrogenase, DH) and thioester reduction and transamination is catalyzed by the bifunctional TamH (thioester reductase, TR, transaminase, TA) which releases the long chain amine (the order of the TamT and TamH reactions is unknown).

We were surprised that the proposed adenylation enzyme for this reaction (AfaA) was located outwith the Tam cluster. Our revisiting of the cluster led us to an ORF with a previously un-ascribed function, TamA, which we hypothesised could fulfil this role. Domain assignment and sequence alignment of the encoded ∼75 kDa protein using BLAST^19^ suggested that it is comprised of two fused domains; an N-terminal adenylation enzyme (ANL, ∼65 kDa)^20^,^21^ and a C-terminal acyl-carrier protein (ACP, ∼10 kDa, Fig. 2 and Fig. S4†).^22^,^23^ Therefore, we hypothesised that TamA catalyses ATP-dependent activation of lauric acid to generate lauryl-adenylate, which it then uses to deliver C12 to the 4′-phosphopantetheine (4′-PP) arm of the C-terminal ACP domain (Fig. 2). This pathway has the advantage over the CoASH-dependent pathway by keeping the fatty acid thioester intermediate covalently protein-bound and siphoning it from the fatty acid pool for subsequent biosynthetic steps. The C12 thioester is predicted to be further modified by dehydrogenation by the FAD-dependent enzyme TamT, that installs the 3, 4 double bond (Table S1†). Finally, the bifunctional TamH is thought to catalyse the unusual reduction and amination to yield a C12 amine. The C-terminal domain, a predicted reductase, initiates the two-step reaction by generating a C12 aldehyde and the N-terminal domain catalyses the amino acid dependent transamination.^24^,^25^ The resultant amine (**3**, Fig. 1) can finally be condensed with the bipyrrole backbone to form tambjamine YP1 (Fig. S2†).

**Fig. 3:**
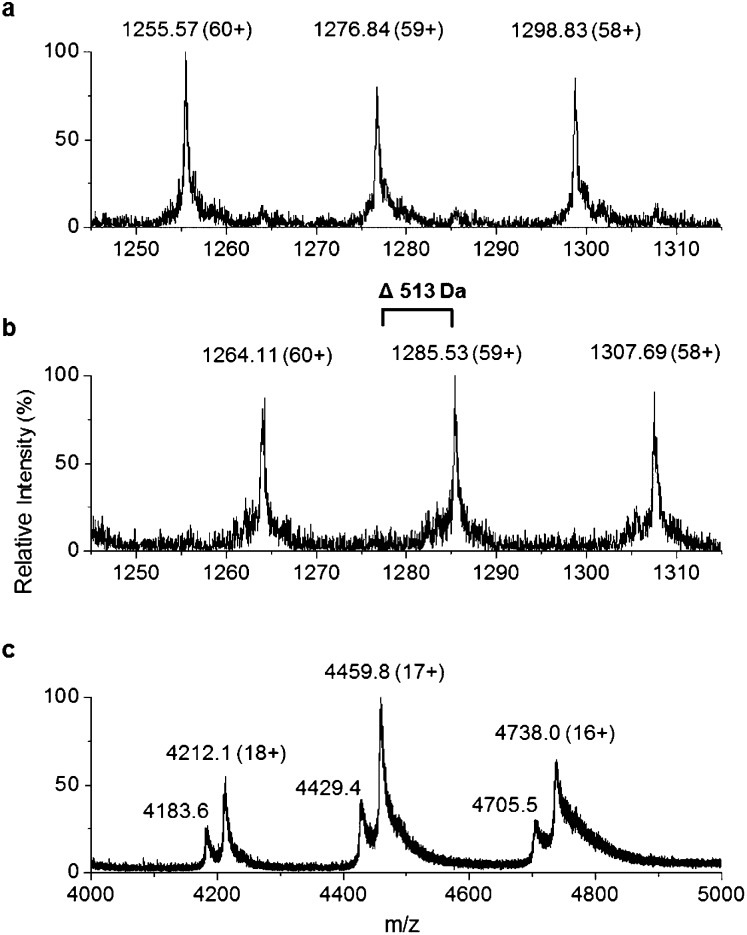
Denaturing ESI-MS over the charge states 60+ to 58+ of (a) *apo*-TamA and (b) *holo*-TamA showing a mass difference of 513 Da. The native mass spectrum (c) of TamA as-purified shows two species for each charge state (18+ to 16+). The higher abundance species is consistent with a TamA:acyl-adenylate bound complex.

Recombinant *P. tunicata* TamA was prepared from *E. coli* with an N-terminal 6xHis affinity tag^26^ (Fig. S5†). It was purified to homogeneity by standard nickel affinity and gel filtration chromatography which showed it to be monomeric (Fig. S5†). The mass (75 271 ± 2 Da) of the purified TamA was determined by denaturing liquid chromatography electrospray ionization – mass spectrometry (LC ESI-MS, Fig. 3a and Fig. S6†). This is consistent with the predicted value (75 270 Da) of the *apo*-protein lacking the 4′-PP post-translational modification on the serine residue of the ACP domain. We employed the commonly used *Bacillus subtilis* Sfp/CoASH system in an effort to convert the *apo*-TamA ACP domain to the *holo*-form.^27^,^28^ However, the expected mass shift that accompanies the addition of a 4′-PP (340 Da) was not observed (Fig. 3b). Instead, the observed mass difference (Δ513 ± 3 Da) is 173 Da larger than anticipated. This mass suggested that the TamA ACP domain had undergone some form of acylation as well as 4′-PP modification. For this to occur in the absence of any fatty acid in the Sfp/CoASH reaction, we hypothesised that the recombinant TamA was isolated with a bound acyl-adenylate. Thereafter, upon 4′-PP modification with Sfp, the TamA ANL domain would catalyse transfer of the acyl chain from the already present acyl-adenylate to the newly-formed *holo*-ACP domain.

To determine if an acyl-adenylate was non-covalently bound in the as-purified TamA, we employed native mass spectrometry that we recently used to observe a BioW/pimeloyl-adenylate complex.^29^Fig. 3c shows the native MS of TamA directly after purification with two species observed over three charge states (18+ to 16+) with nominal masses of 75 800 and 75 280 Da. The average mass difference of these species (∼520 Da) is close to the calculated mass of the expected C12 lauryl-adenylate (529 Da). Although this native MS method is not accurate enough to determine the exact mass of the bound molecule^30^ it does confirm the presence of a non-covalently bound species. We attempted to remove the acyl-adenylate from the protein active site by incubating it overnight with CoASH and then repeating the native MS. However, there was no change in the mass spectrum suggesting that CoASH is not a substrate for the TamA ANL.

Based on the YP1 structure we expected TamA to utilise C12 fatty acids as substrates. However, the mass we observed of the 4′-PP modified and acylated full length TamA was not consistent with a C12 thioester attached to the ACP domain. Attachment of a C12 fatty acid should result in a mass increase of 522 Da (182 Da larger than 4′-PP) but the mass shift we observed was 513 Da (173 Da larger than 4′-PP, Fig. 3b). Since this does not correspond to the mass of any unsaturated fatty acids, we postulated that this species could be *holo*-TamA modified by a mixture of fatty acid thioesters. The small mass shifts expected from fatty acids of similar length may not resolve due to the large size and high charge state of the full length protein.

**Fig. 4:**
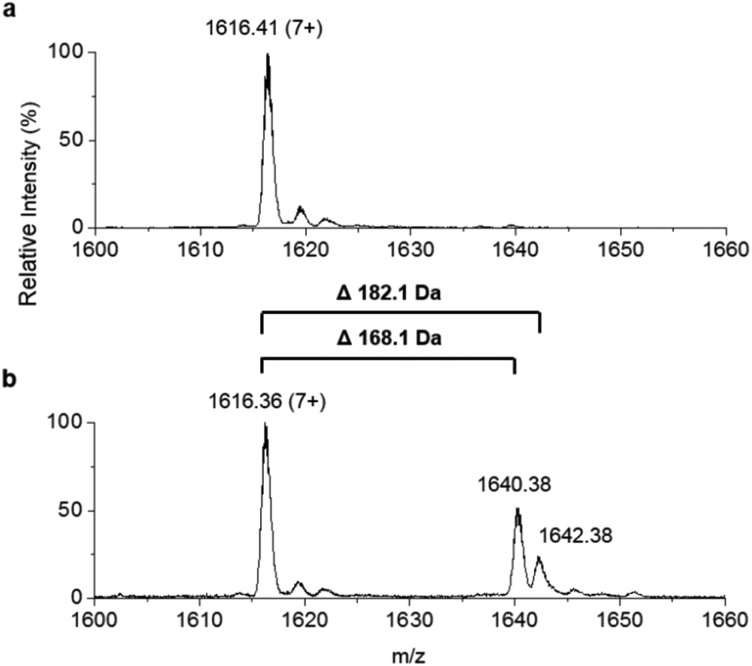
Denaturing ESI-MS analysis of the 7+ charge state of (a) *holo*-TamA ACP domain and (b) *holo*-TamA ACP after incubation with *apo*-TamA.

To improve the resolution of the MS analysis, we sought to capture the fatty acid moiety on a smaller ACP protein. So, guided by our domain analysis, the predicted ACP portion of the enzyme was expressed and purified (Fig. S7†). LC ESI-MS analysis revealed that the ACP domain was isolated in the *apo*-form (predicted mass = 10 967.5 Da, observed mass = 10 967.1 ± 0.2 Da, Fig. S8†). Quantitative conversion to the *holo*-ACP form was achieved with the Sfp/CoASH system (predicted mass = 11 307.8 Da, observed mass = 11 307.6 ± 0.1 Da, Fig. 4a). This *holo*-ACP domain was subsequently incubated with full length, as-purified *apo*-TamA (with acyl-adenylate(s) bound) to attempt the thiolation reaction *in trans* – in effect an intermolecular, *trans*-thioesterification. LC ESI-MS analysis of this reaction revealed the appearance of two new acyl-ACPs. These correspond to the addition of 168.1 ± 0.2 and 182.1 ± 0.4 Da respectively (Fig. 4b) and these mass differences are consistent with the attachment of a C11 and C12 fatty acid to the *holo*-ACP (predicted mass, 168.3 and 182.3 Da respectively). Although the presence of a C11 fatty acid is unexpected since only the C12 form of YP1 has been reported for *P. tunicata*, other organisms produce tambjamines with varying acyl chain lengths.^2^,^4^ In the *E. coli* host we presume the enzyme has picked up the C11 and C12 fatty acids from the endogenous fatty acid pool. This data also shows that the catalytic TamA ANL domain is able to transfer acyl chains to an isolated ACP.

**Fig. 5:**
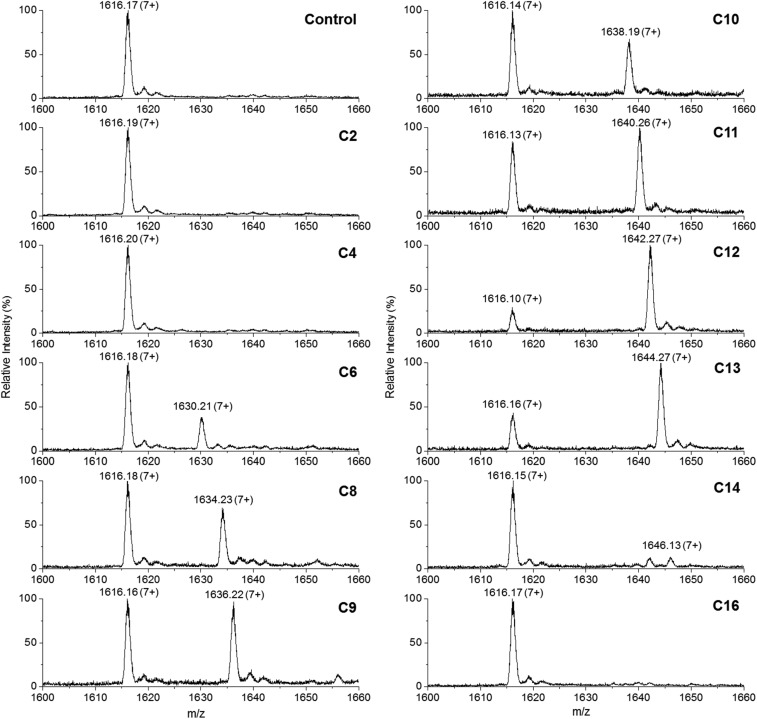
Denaturing ESI-MS analysis of the 7+ charge state of the *holo*-TamA ACP domain after incubation with *holo*-TamA, Mg^2+^, ATP and fatty acids ranging in length from C2–C16. The values of the deconvoluted masses are described in Table S2.†

This convenient transfer reaction was used to determine the fatty acid chain length specificity of the ANL domain. *Holo*-TamA produced after 4′-PP modification of the as-purified TamA was incubated with different fatty acids, Mg^2+^, ATP and the *holo*-TamA ACP domain. LC ESI-MS analysis of the ACP domain showed the extent of acylation for a range of fatty acids between C2 and C16 (Fig. 5, Table S2†). The data reveals that TamA is able to utilise fatty acids from C6–C13 (the C14 form is barely detectable) however, conversion to the acylated form is highest for C12. The efficiency of the reaction drops off substantially outwith the C12–C13 substrates suggesting a very specific substrate pocket in the ANL active site. In order to test the specificity of the ANL domain towards other ACPs, *E. coli* ACP (Fig. S9†) was also used in the assay in place of the standalone TamA ACP domain. However, TamA is unable to transfer the C12 fatty acid to *E. coli* ACP suggesting a highly specific recognition between the ANL and ACP domains (Fig. S10†). It would be interesting to explore this specificity further with a range of ACPs from different species.^22^,^23^

With convincing data supporting acylation of TamA we wanted to clearly assign the site of 4′-PP attachment. The unusual TamA ACP domain contains two conserved DSV 4′-PP motifs^22^,^23^ but the TamA sequence analysis suggests that the second DSV motif (residues 621–623) is the most likely site of modification. *Holo*-TamA was denatured and digested with trypsin and the resulting peptide mixture was analysed by ESI-MS. Masses for the acylated 4′-PP peptides were not observed, potentially due to the instability of the thioester bond under the denaturation conditions. However, a peptide mass consistent with the 4′-PP on residue S622 was observed and subjected to MS/MS analysis. A combination of 4′-PP ejection^31^ and peptide sequencing confirmed S622 as the site of the 4′-PP modification (Fig. S11†).

Diffraction quality crystals of recombinant TamA have not been obtained so we used homology modelling to give some insight into the TamA structure. The ANL domain displays highest similarity (∼30%) to fatty acid AMP-ligases (FAALs, Fig. S12†)^32^–^35^ which activate fatty acids for transfer to the terminal 4′-PP thiol of a separate ACP. FAALs are homologous to FACLs but are unable to use free CoASH as a substrate. CoASH is in fact blocked from binding by an insertion loop common to all FAALs, that acts as a gatekeeper to the active site, released upon ACP binding.^35^ However, the structural model could not be built using these enzymes as there are no reported structures of either a FAAL-ACP fusion or a FAAL in complex with its cognate ACP.

Nonetheless, TamA is a member of the Type I fold of the ANL enzyme superfamily which includes firefly luciferases, acyl-CoA synthetases and the adenylation domains of nonribosomal peptide synthetases (NRPS). Since it shares ∼20% sequence identity with both NRPS ANL and peptide carrier protein (PCP) domains a model of the TamA structure was built with Phyre2 36 software using four homologous NRPS enzyme sequences (Fig. S13 and 14†).^37^–^41^ The model displays the canonical Type I ANL fold as well as the recognizable four-helical bundle of the carrier protein domain with the 4′-PP modification on S622 located.^22^,^23^ This working model allows us to identify residues potentially involved in substrate binding and catalysis.

## Conclusions

In conclusion, we have provided evidence that allows us to assign an important function to the previously uncharacterized TamA from the tambjamine biosynthetic pathway. Throughout isolation the recombinant enzyme stabilizes a mixture of C11 and C12 fatty acid adenylates. We have also shown that the ANL domain is able to catalyse adenylation and thiolation reactions on its fused ACP partner as well as transferring fatty acids of various chain lengths to its separate ACP domain. Since C12 is the reported chain length of tambjamine YP1, we postulate that TamA initiates the synthesis of the essential amine chain. With the acylated forms of TamA in hand, we can now explore their interactions with the subsequent enzymes, TamT and TamH, in the biosynthetic pathway. Since TamA links fatty acid and pyrrole biosynthesis, our work lays the foundations for engineering this enzyme to guide production of novel tambjamines with enhanced biological activity.

## Conflicts of interest

There are no conflicts to declare.

## Supplementary Material

Supplementary information
