# Supplementary material for: The carbon chain-selective adenylation enzyme TamA: the missing link between fatty acid and pyrrole natural product biosynthesis
Source: Org Biomol Chem. 2018 Mar 22;16(15):2735–40. doi: 10.1039/c8ob00441b (PMC5939613; doi:10.1039/c8ob00441b)
Supplement: Supplementary file 1 [file OB-016-C8OB00441B-s001.pdf]

Electronic Supplementary Material (ESI) for Organic & Biomolecular Chemistry.
This journal is © The Royal Society of Chemistry 2018

The carbon chain-selective adenylation enzyme TamA: the missing link between fatty acid and pyrrole natural product biosynthesis.

Supplementary Information

Piera M. Marchetti, Van Kelly, Joanna P. Simpson,

Mairi Ward and Dominic J. Campopiano*

Primary data files can be found at:

http://dx.doi.org/10.7488/ds/2327

Materials and Methods

Cloning, Expression and Purification of TamA and TamA ACP: TamA was cloned from *Pseudoalteromonas tunicata D2* genomic DNA into the pEHISTEV vector[1] using NcoI and XhoI restriction sites to yield recombinant TamA with a Tobacco Etch Virus (TEV) protease cleavable N-terminal 6xHis-tag. The TamA ACP was cloned from the TamA plasmid by the same method. The plasmids were used to transform *E. coli* BL21 DE3 cells (Novagen) which were grown in 2 L of LB media with kanamycin. Once the cultures reached an OD_{600} ~ 0.8 they were induced to express the gene using 0.1 mM IPTG and left shaking at 16°C overnight. Purification of the proteins was carried out on ice or at 4°C. Harvested cells were resuspended in binding buffer (50 mM Tris pH 8, 250 mM NaCl, 10 mM Imidazole and 10% glycerol) and lysed by sonication. The cell lysate was clarified by centrifugation at 24,000 g for 30 min and filtered through a 0.45 µm filter. The cell-free extract was loaded onto a 1 mL HisTrap nickel affinity column (GE Healthcare). The column was washed with binding buffer for 20 column volumes, then the protein eluted with an imidazole gradient (10 to 500 mM) over 30 column volumes. Protein containing fractions were pooled, mixed with 1 mg of TEV protease and dialysed against dialysis buffer (50 mM Tris pH 8, 250 mM NaCl and 10% glycerol) for 1 hour. The HisTrap nickel affinity column was repeated, this time collecting the tag-free protein in the flow-through and column wash. Finally, the protein was concentrated to 2 mL and loaded on a 120 mL Superdex S200 or Superdex S75 gel filtration column with gel filtration chromatography (GFC) buffer (50 mM Tris pH 8, 250 mM NaCl, 1 mM DTT and 10% glycerol). Purification was monitored by SDS-PAGE.

Sfp Reaction: TamA and TamA/ACP were 4'-phosphopantetheine (4'-PP) modified during the purification process. After the TEV protease cleavage and dialysis, the TamA or TamA ACP protein was concentrated and added to a 10 mL reaction containing 10 µM Sfp, 10 mM $MgCl_2$ and 500 µM CoASH in dialysis buffer. This reaction was left shaking for 16h at 4 °C. The protein purification was subsequently continued in the same way with the second nickel affinity chromatography step removing the Sfp along with the TEV protease.

Protein LC-ESI-MS: 5 µL of 10 µM protein was injected onto a Phenomenex C4 3.6u column. Protein was eluted with 0.1% formic acid with a gradient of 5 to 95% acetonitrile and 0.1% formic acid. Mass spectra were obtained using electrospray ionisation (ESI) coupled to a Synapt G2 Q-TOF (Waters) with the source set at 120 °C, backing pressure 2 mbar, and sampling cone voltage of 54 V. Protein spectra are presented after subtraction using the MassLynx V4.1 software. Peak *m/z* annotations were extracted from smoothed and centroided data. The centroided data was also used in the component algorithm to extract the protein average masses.

Native Protein MS: TamA was desalted into 300 mM ammonium bicarbonate using a P6 Micro Bio-Spin column and diluted to 10 µM. Protein was infused by nano-electrospray ionisation (nano-ESI) using a TriVersa NanoMate (Advion) set at 1.7 kV and 0.8 psi. ESI was coupled to a Synapt G2 Q-TOF (Waters) with the source set at 80 °C, backing pressure increased to 5 mbar, and sampling cone voltage of 200 V. The spectrum was averaged over 4 min of acquisition data and presented without further data processing. Peak *m/z* annotations were extracted from smoothed and centroided data using Waters MassLynx V4.1 software. The centroided data was also used in the component algorithm to extract the protein average masses.

Fatty Acid Transfer Assay: *Holo*-TamA ACP was mixed 1:1 with *apo*-TamA at a concentration of 10 µM of each protein in GFC buffer. These were incubated for 16h at room temperature before being

subjected to LC ESI-MS. To test the transfer of other fatty acids, 10 µM *holo*-TamA ACP was incubated with 10 µM *holo*-TamA, 1 µM fatty acid (from a 10 µM stock in DMSO), 5 mM ATP, 10 mM $MgCl_2$ in GFC buffer for 20h at room temperature. The reactions were then subjected to LC ESI-MS.

Expression and Purification of *E. coli* ACP: *E. coli* ACP in pET28a with no tag and *B. subtilis* Sfp in pET-ACYC with no tag were used to transform *E. coli* BL21 DE3 cells (Novagen) which were grown in 1 L of LB media with kanamycin and chloramphenicol. Once the cultures reached an OD_{600} ~ 0.8 they were induced to express the genes using 0.5 mM IPTG and left shaking at 18°C overnight. Purification of the protein was carried out on ice or at 4°C. Harvested cells were resuspended in binding buffer (50 mM tris-HCl pH 7.5, 100 mM NaCl, 1 mM DTT) and lysed by sonication. The cell lysate was clarified by centrifugation at 24,000 g for 30 min and filtered through a 0.45 µm filter. The cell-free extract was loaded onto a 1 mL HiTrap Q anion exchange column (GE Healthcare). The column was washed with binding buffer for 5 column volumes, then the protein eluted with an NaCl gradient (100 to 1000 mM) over 20 column volumes. Protein containing fractions were pooled, concentrated to 2 mL and loaded on a 120 mL Superdex S75 gel filtration column with GFC buffer (20 mM HEPES pH 7.5, 100mM KCl). Purification was monitored by SDS-PAGE.

Peptide digest and MS: 50 µg of *holo*-TamA was denatured by addition of 8 M urea to a final concentration of 6 M before incubation at room temperature for 2 hours. The protein was subsequently reduced with DTT added to a final concentration of 5 mM and further incubated at room temperature for 30 mins. The samples were alkylated with iodoacetamide at a final concentration of 10 mM at room temperature in the dark for 30 mins. This reaction was quenched by adding more DTT to a final concentration of 10 mM and incubating at room temperature for 30 mins. The sample was then diluted to a urea concentration of less than 1 M with 50 mM ammonium bicarbonate. Finally, 1 µg of trypsin was added to the sample and this was incubated at 37 °C overnight. The samples were cleaned using C18 100 µL ziptips and washed in 0.2 % formic acid. Peptides were eluted in 60 % ACN with 0.2 % formic acid. The resulting peptide mixtures were analysed using a Bruker solariX XR 12T. The solariX was operated in positive ion mode and protein was infused using nano-ESI on a TriVersa nanomate (Advion). Data was acquired over an *m/z* range of 200 – 5000 with an average of 30-50 scans per sample and an accumulation time of 0.2 seconds. Spectra were processed and calibrated with DataAnalysis version 4.2. The $[M+3H]^{3+}$ ion containing 4'-PP modification was isolated for further MS/MS analysis. In order to isolate this ion, the accumulation time was increased to 2 seconds and an average of 50 spectra were acquired with a 2 Da window. The resulting product ion spectrum of $[M+3H]^{3+}$ was then analysed for y and b ions corresponding to a 4'-PP modification using prositeLite.

Supplementary Figures

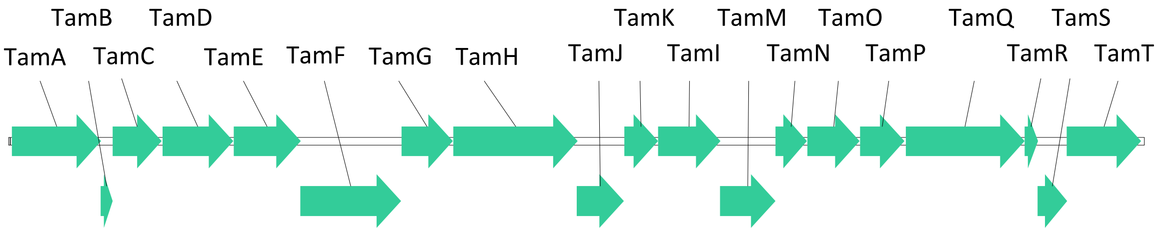

Figure S1: Organisation of the 19 open reading frames (ORFs) of the tambjamine YP1 gene cluster[2]. Table S1 gives the Genbank code and predicted function for each gene.

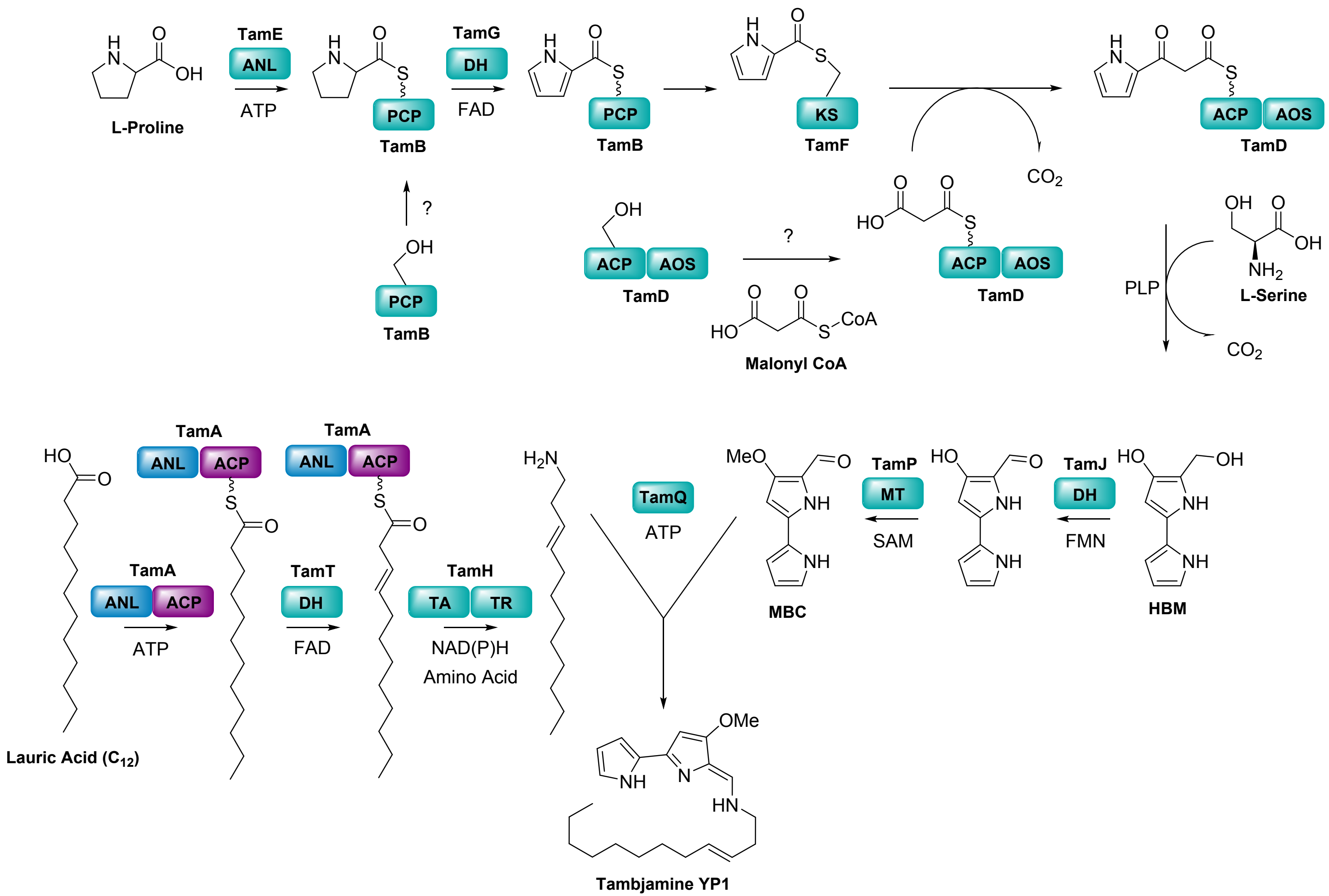

Figure S2: Proposed tambjamine YP1 biosynthetic pathway, adapted from Kjelleberg and coworkers[2]. Roles for 11/19 proteins from the operon are included. Protein abbreviations: ACP - acyl carrier protein, ANL - adenylation enzyme, AOS - α-oxoamine synthase, DH - dehydrogenase, KS - ketosynthase, MT - methyltransferase, PCP - peptidyl carrier protein, TA - transaminase, TR - thioester reductase. Small molecule abbreviations: HBM - 4-hydroxy 2,2’-bipyrrole 5-methanol, MBC - 4-methoxy 2,2 bipyrrole carbaldehyde.

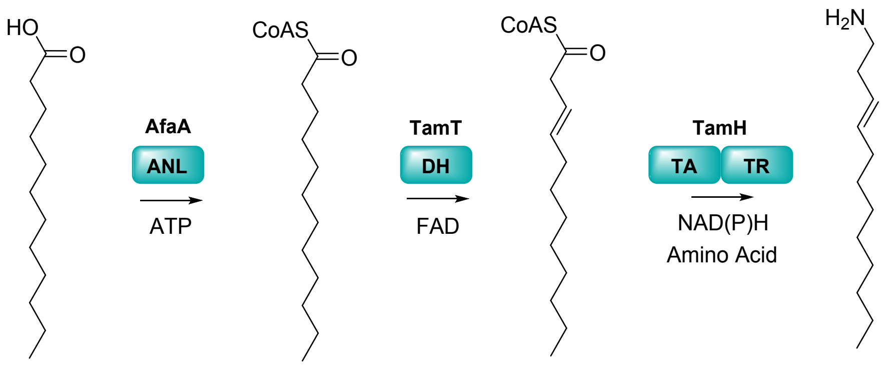

Figure S3: Pathway to amine formation suggested by Kjelleberg and coworkers[2] initiated with the adenylation of lauric acid by AfaA and its transfer to Coenzyme A (CoASH).

```
  1
GAMECEASSL IDLLAFDARN KPSQEVFRFV SDNGESEASY DYQTLSQEIS RIAIGLQALI  58
KTSHNQDQAL IVLPQGVQFV TAFYGCMAAN VIAVPSFPPK SQLQIERLQF AITDLGNPIV 118
ITNRDILPKL QEHIALDSVR WLLIEDLASV IAQPLSDFRT HEHSIALLQY SSGTTGKPKG 178
VIITNQNIME NSELIRQSFG HKEDHTRMML WLPPHHDMGL VGGVMQGVYT GYPTLLMPTD 238
LFLRSQYRWL KAVSDYRATT TGAPNFAYEL AVKNIRESRL AELDLSSLEN LFCGAEPINS 298
HSINQFLDKF APCGLKPEAF LPCYGMAEAT LMVSGKPHGQ QYKQLCIDEP LLKHGMVKPL 358
NTPNAHSLWL VSSGVVHSSL QARIVNPETG TEVAQGQVGE IWLQGSSISP GYWQDAERTA 418
INFGLPLAGY EETFHRTGDL GFYHQDELFI TGRLKEVVII RGANFYPQDL EYETTLAFPE 478
LNNCRSAAFS VPKEGKEQLI MAIEVPRNVT EFNQYAKILN GRLVERFGIR ADIILFLPRK 538
TIKITSSGKL QRVAIKKAYE EQQLPVYFQY QLQGEQIAPR EVSLDISNQD SVAKWLVARV 598
SELTGVAIAQ ISEHEPLTNV GLDSVLAMEI LFRLEQQTGV YLAPDVLYSC NTPSLLAEQI 658
IKVAGNVAEK ELNLSC                                                 674
```

Figure S4: The UNIPROT code A4C5W5 was used to design expression of TamA. Small changes were made (addition of GA at the beginning of the sequence and E2Q) during cloning. Amino acid sequence of TamA with the predicted (BLASTP)[3] adenylation (ANL) domain in blue and the acyl carrier protein (ACP) domain in purple with short connecting “linker” sequence in between (GEQIAPREVS) . In bold, underlined are the two DSV motifs which could be the attachment sites for 4’-phosphopantetheine (4’-PP). The serine in red (S622) is predicted to be the modification site.

a

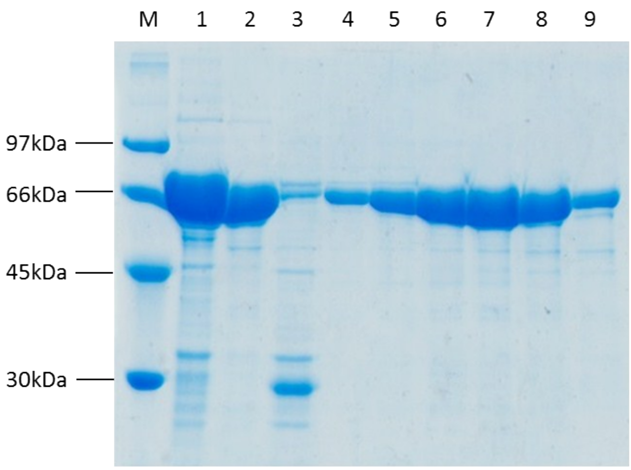

b

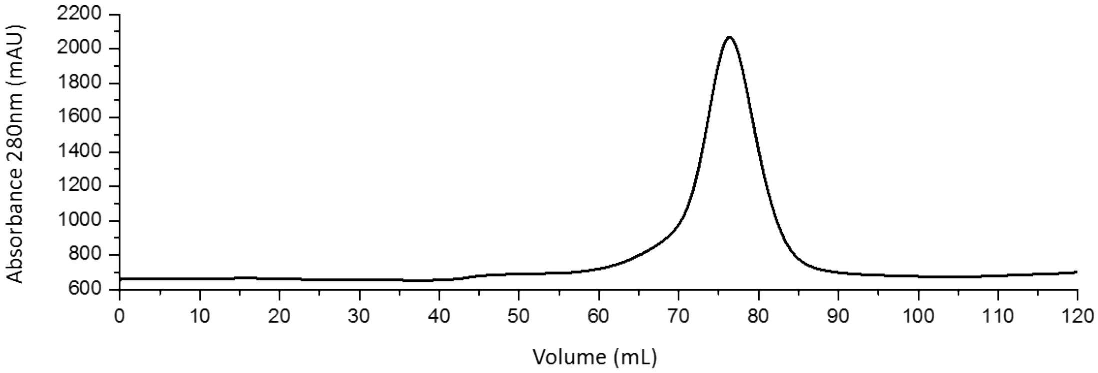

Figure S5: (**a**) 10% SDS-PAGE gel of TamA purification steps. M: GE Healthcare LMW Marker, Lane 1: TamA after nickel affinity purification, Lane 2: flow-through of second nickel affinity purification after TEV cleavage of the 6xHis-tag, Lane 3: elution from nickel affinity after TEV cleavage, Lanes 4-9: fractions from the Superdex HR S200 (GE Healthcare) gel filtration chromatography (3 mL fractions from 67-85 mL), (**b**) chromatogram of TamA isolation by gel filtration chromatography.

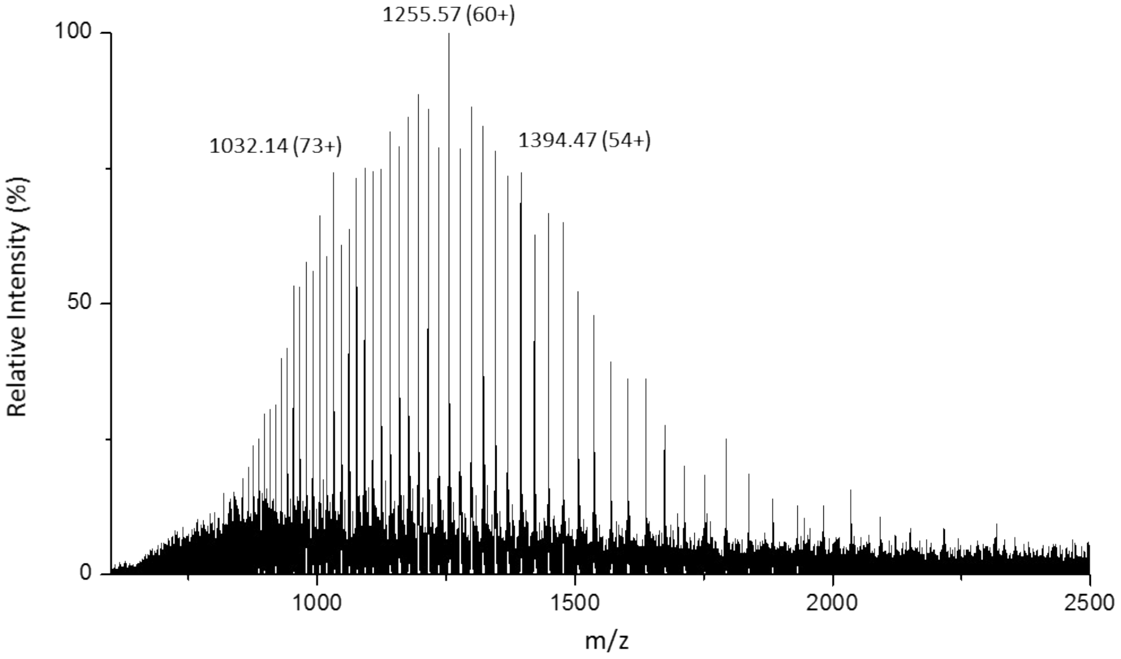

Figure S6: Denaturing liquid chromatography - mass spectrometry (LC-MS) of *apo*-TamA as purified. This charge state distribution is consistent with a deconvoluted mass of 75271±2 Da.

a

576
GAMAPREVSL DISNQ**DSV**AKW LVARVSELTG VAIAQISEHE PLTNVGL**DSV** 623
LAMEILFRLE QQTGVYLAPD VLYSCNTPSLL AEQIIKVAGN VAEKELNLSC 674

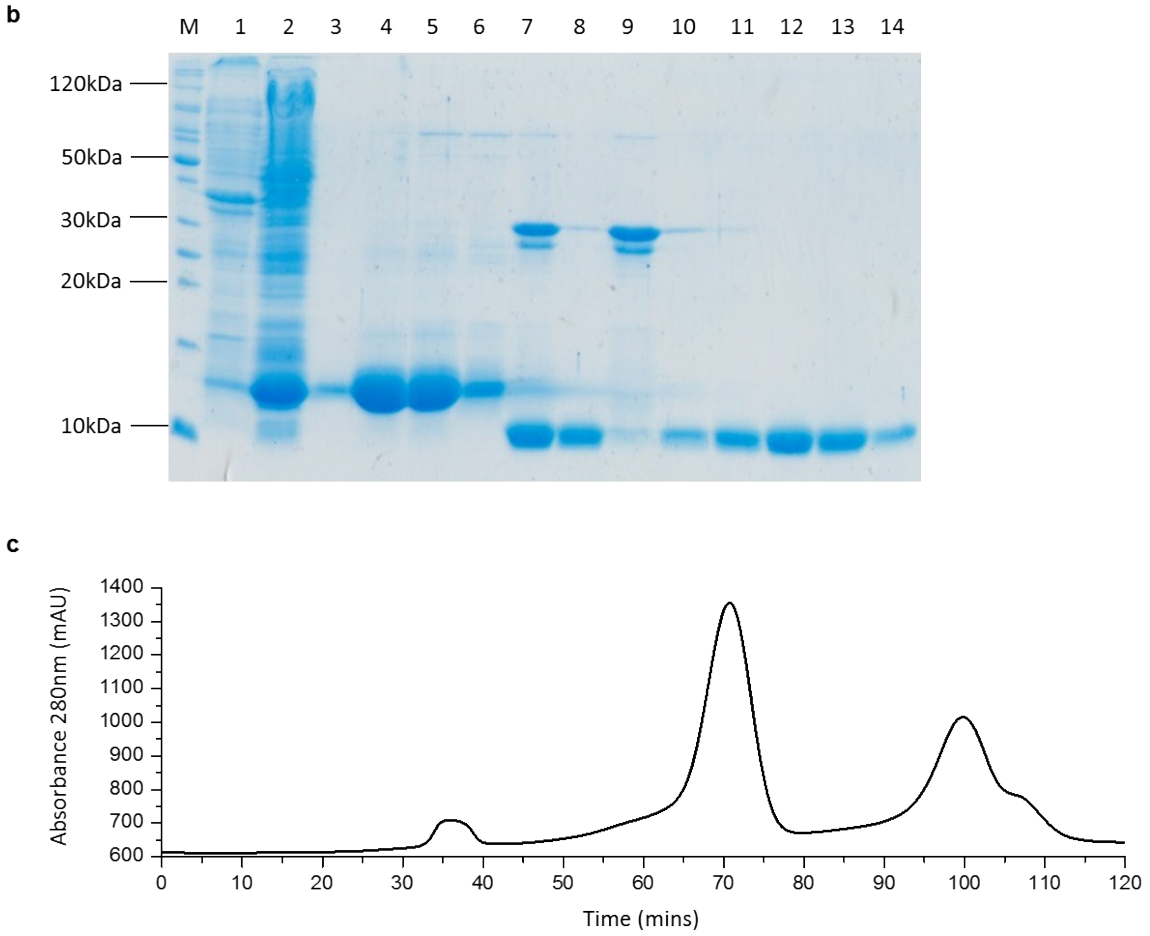

Figure S7: (**a**) Sequence of the recombinant TamA acyl carrier protein (ACP) domain beginning at residue A576. Three amino acid residues (GAM) were added to the beginning of the sequence as a result of His-tag addition/TEV cleavage, followed by the residual linker sequence (APREVS). In bold are the two DSV motifs where 4'-phosphopantetheine (4'-PP) attachment sites could be, the serine in red is the one predicted to be modified, (**b**) 15% SDS-PAGE gel of TamA ACP purification steps, M: PageRuler Plus marker, Lane 1: insoluble fraction of cell lysate, Lane 2: soluble fraction of cell lysate, Lanes 3-6: fractions of nickel affinity purification, Lane 7: TamA ACP after TEV cleavage of the 6xHis-tag, Lane 8: flow-through of second nickel affinity purification, Lane 9: elution from nickel affinity after TEV cleavage, Lanes 10-14: fractions from superdex S75 gel filtration chromatography (3 mL fractions from 64-76 mL), (**c**) chromatogram from superdex S75 gel filtration chromatography of TamA ACP.

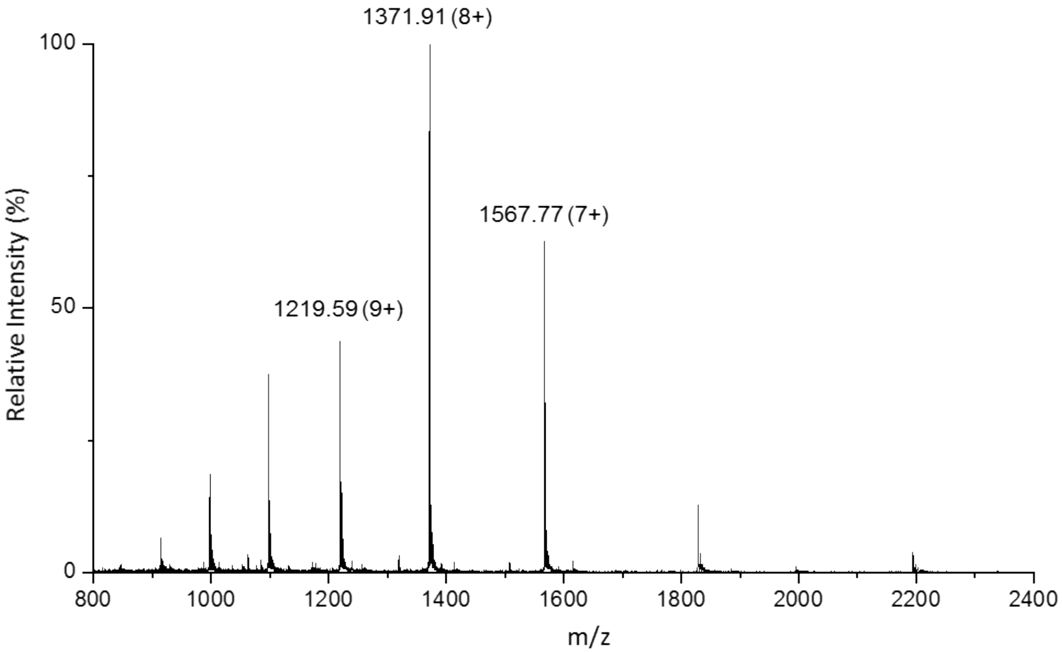

Figure S8: Denaturing liquid chromatography electrospray ionisation mass spectrometry (LC ESI-MS) of *apo*-TamA acyl carrier protein (ACP). This charge state distribution is consistent with a deconvoluted mass of 10967.1±0.2 Da.

a

MSTIEERVKK IIGEQLGVKQ EEVTNNASFV EDLGA**DSL**DT VELVMALEEE 50
FDTEIPDEEA EKITTVQAAI DYINGHQA 68

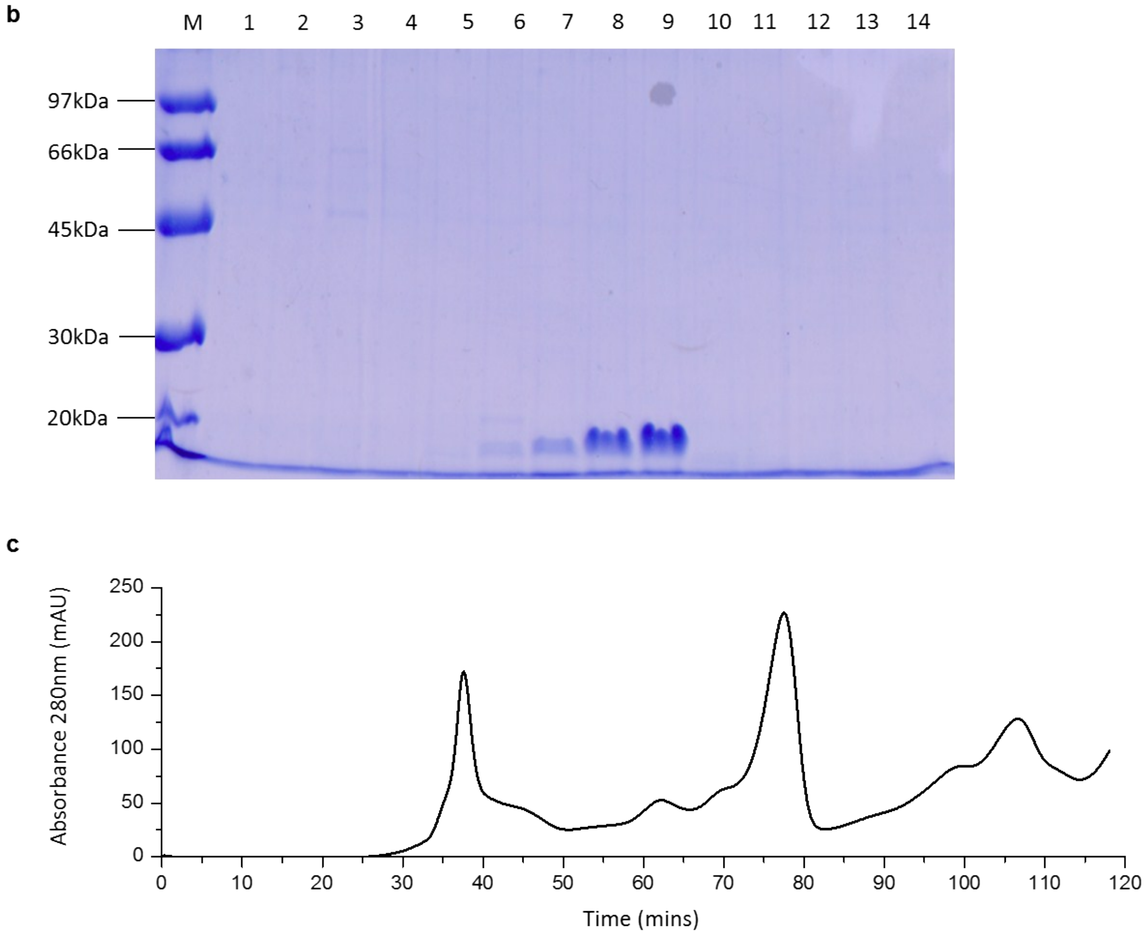

Figure S9: (**a**) Sequence of the recombinant *E. coli* acyl carrier protein (ACP) domain with phosphopantetheine motif in bold, (**b**) 15% SDS-PAGE gel of *E. coli* ACP superdex S75 gel filtration chromatography, M: GE Healthcare LMW Marker, Lane 1: 30-33 mL, Lane 2: 36-39 mL, Lanes 3: 42-45 mL, Lane 4: 48-51 mL, Lane 5: 54-57 mL, Lane 6: 60-63 mL, Lane 7: 66-69 mL, Lane 8: 72-75 mL, Lane 9: 78-81 mL, Lane 10: 84-87 mL, Lane 11: 90-93 mL, Lane 12: 96-99 mL, Lane 13: 102-105 mL, Lane 14: 108-111 mL, (**c**) chromatogram from superdex S75 gel filtration chromatography of *E. coli* ACP.

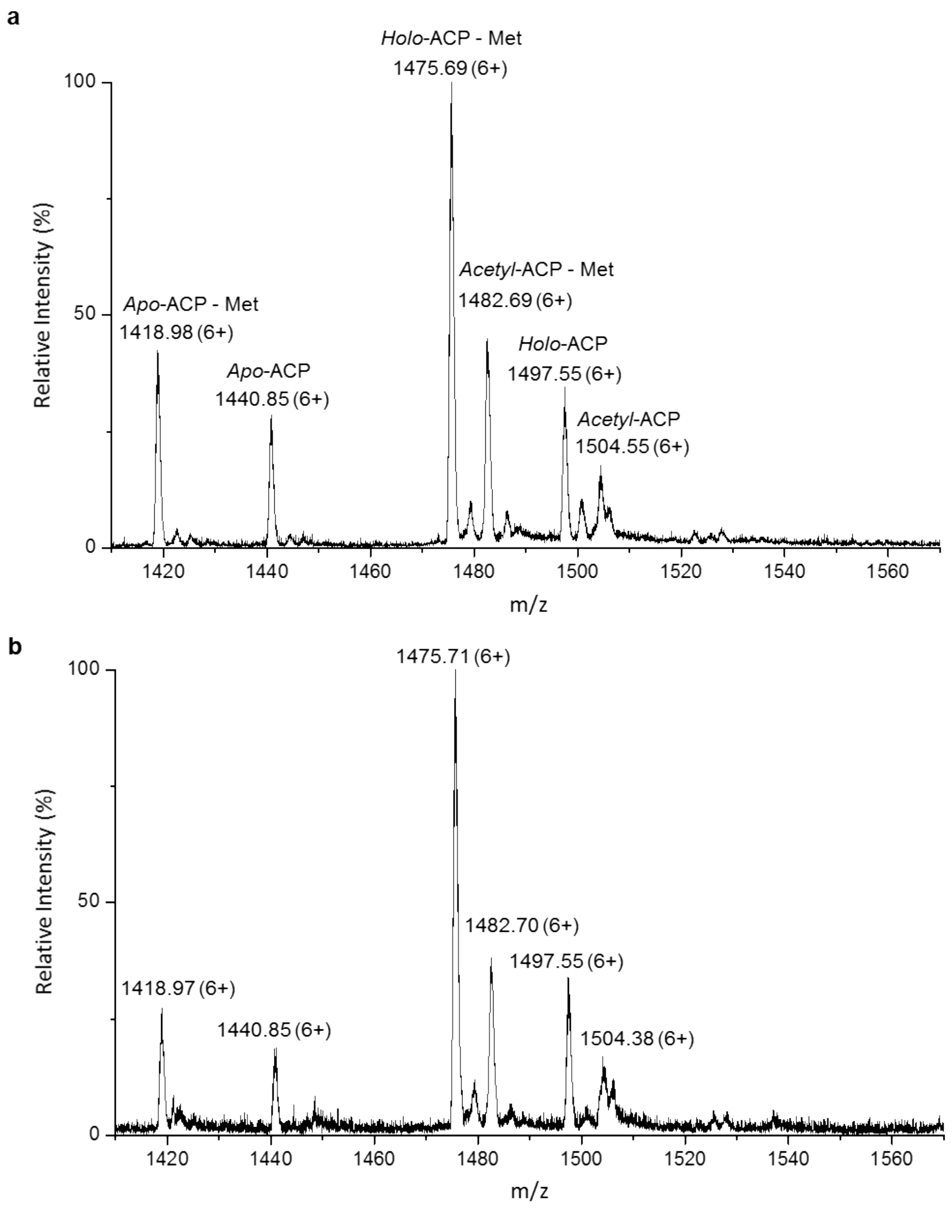

Figure S10: Denaturing liquid chromatography electrospray ionisation mass spectrometry (LC ESI-MS) of the 6+ charge state of (**a**) *E. coli* ACP co-expressed with *B. subtilis* Sfp showing apo and holo- and acetyl-forms of the protein with and without the N-terminal Methionine and (**b**) *E. coli* ACP co-expressed with *B. subtilis* Sfp after incubation with TamA, C12 fatty acid, Mg^{2+} and ATP. This shows no change in the spectrum suggesting that TamA is unable to use *E. coli* ACP as a substrate.

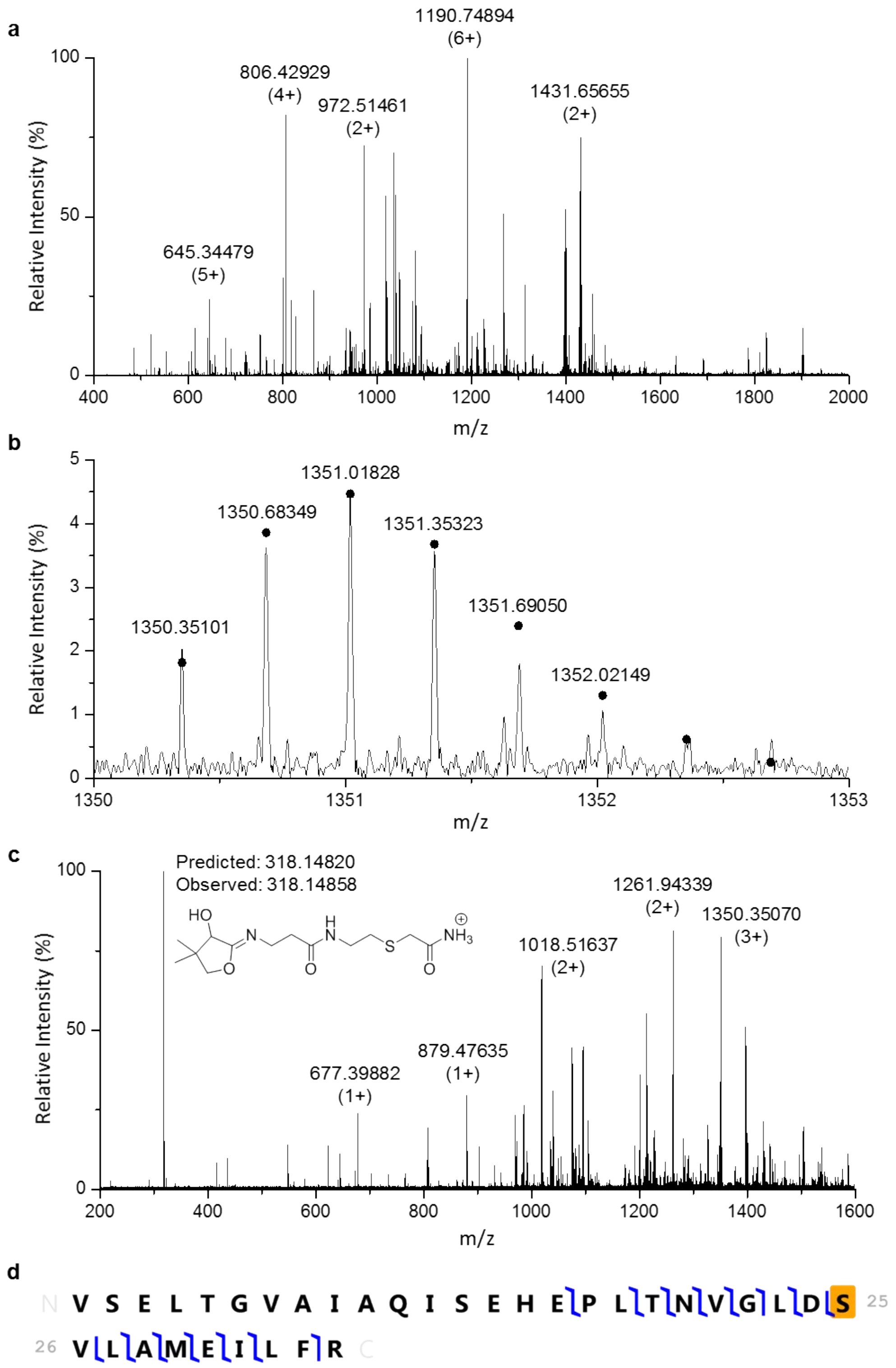

Figure S11: Identification of TamA S622 as the site of 4'-phosphopantetheine (4'-PP) attachment. (**a**) Fourier transform ion cyclotron resonance (FT-ICR) ESI-MS analysis of a tryptic digest of *holo*-TamA showing all peptide peaks and (**b**) zoom in of the 3+ ion series corresponding to the 4'-PP- peptide (sequence VSEL…..ILFR). The black dots show the predicted masses for that peptide. The MS/MS spectrum (**c**) of the 4'-PP modified peptide peak from (**b**) gives a mass corresponding for 4'-PP ejection (predicted mass: 318.14820 Da, observed mass: 318.14858 Da). PrositeLite software analysis of the observed z and b ions from peptide sequence (**d**) confirms that the 4'-PP modification is located on residue S622 (highlighted in orange).

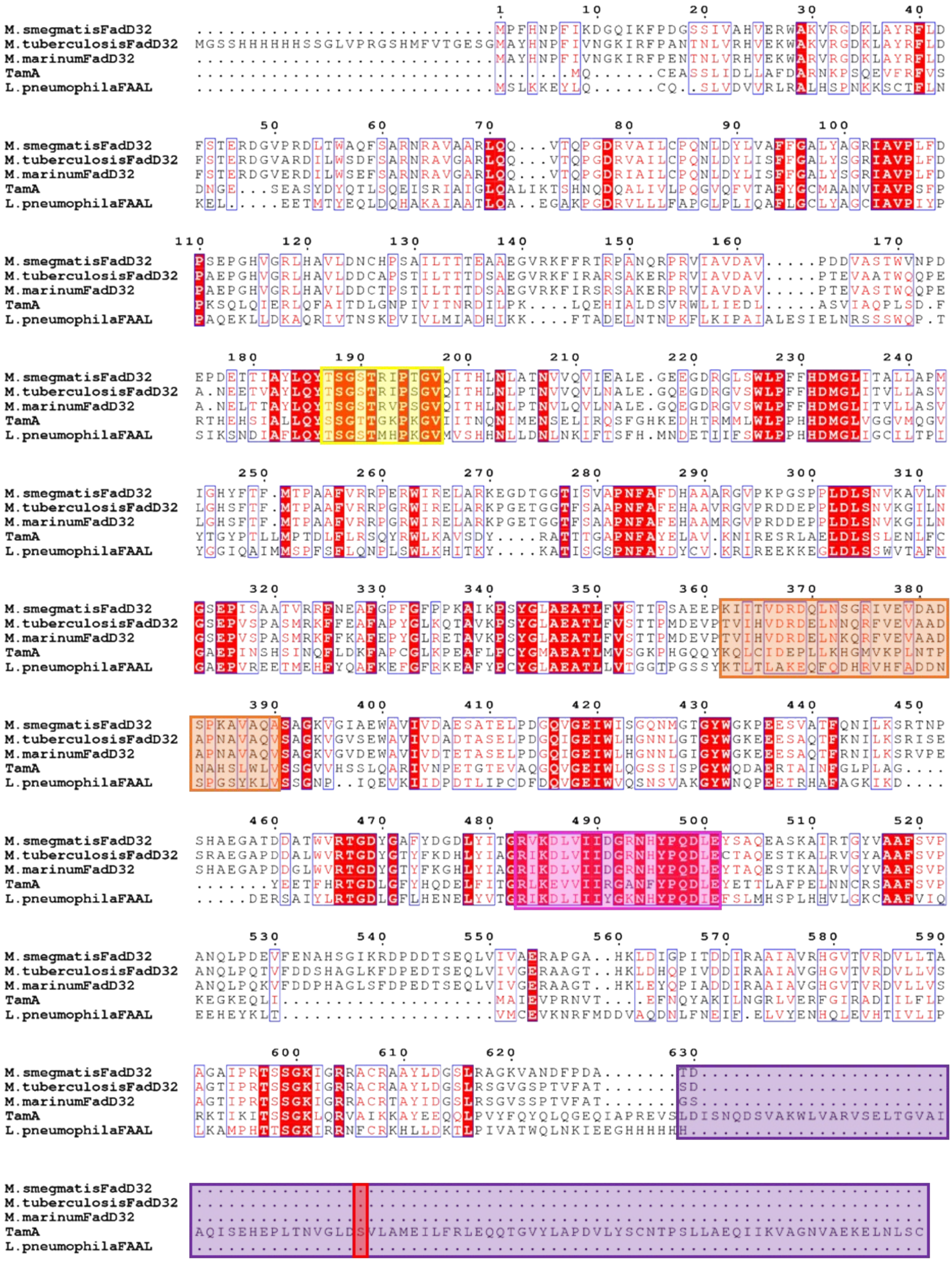

Figure S12: Alignment of TamA with its closest homologues in the PDB, all of which are fatty acid-AMP ligases (FAALs): *M. smegmatis* FadD32 (5D6J), *M. tuberculosis* FadD32 (5HM3), *M. marinum* FadD32 (5EY9) and *L. pneumophila* FAAL (3KXW). Highlighted are the conserved ATP binding P-loop (yellow), the FAAL insertion loop (orange), the hinge region (pink) and the predicted ACP domain (purple) with its conserved serine residue (S622, red).

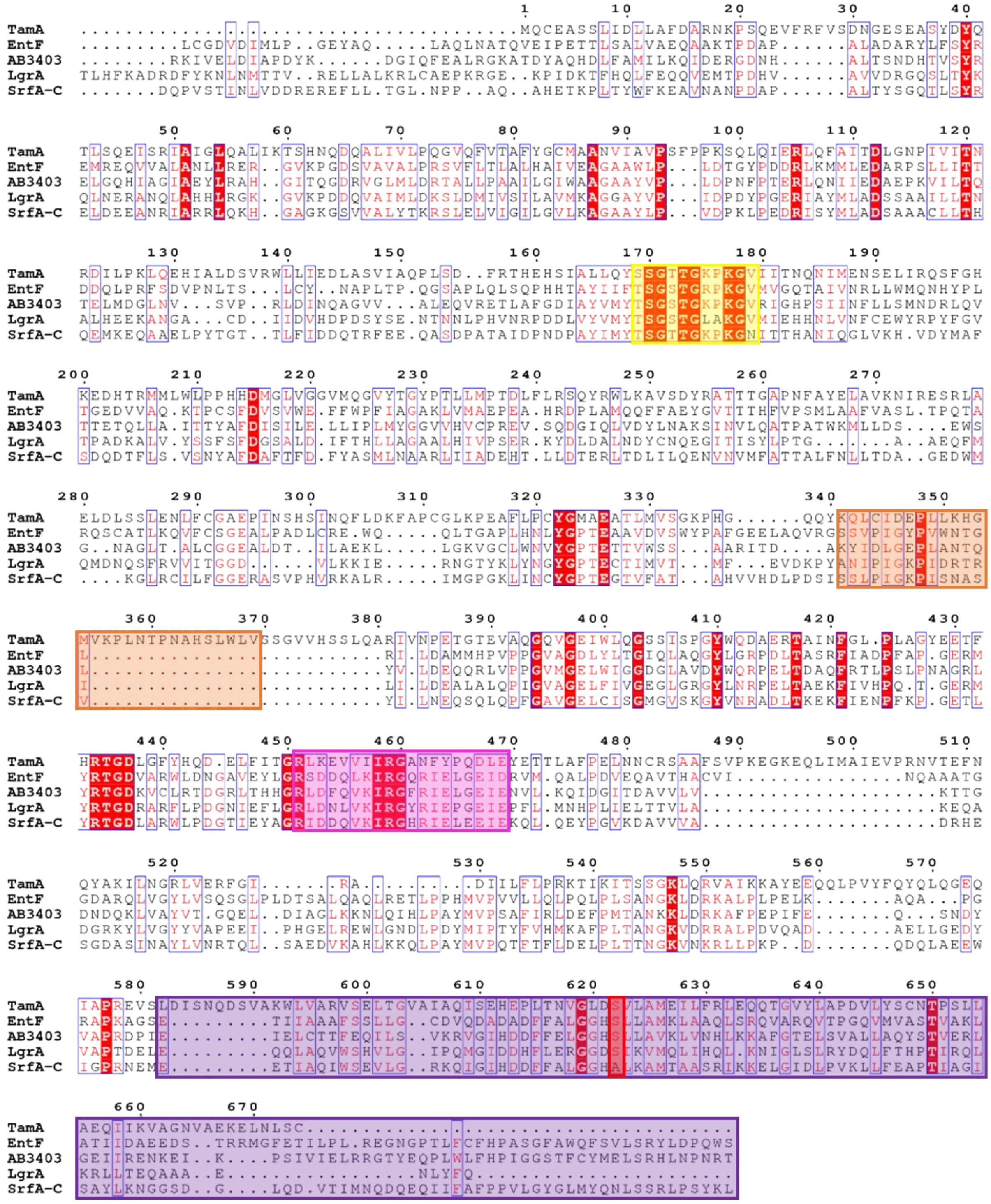

Figure S13: Alignment of the TamA amino acid sequence with sequences from parts of four NRPS proteins. The NRPSs contain peptide carrier protein (PCP) domains. Six crystal structures of these four proteins EntF (PDB codes: 5T3D, 5JA2), AB3403 (4ZXI), LgrA (5ES8) and SrfA-C (5U89, 2VSQ) were used in the production of the TamA structure model. Highlighted are the conserved ATP binding P-loop (yellow), the FAAL insertion loop (orange), the hinge region (pink) and the predicted carrier domain (CP, purple) with its conserved serine residue S622 (red, this is missing in SrfA-C since the 4'-PP site of the PCP has been mutated).

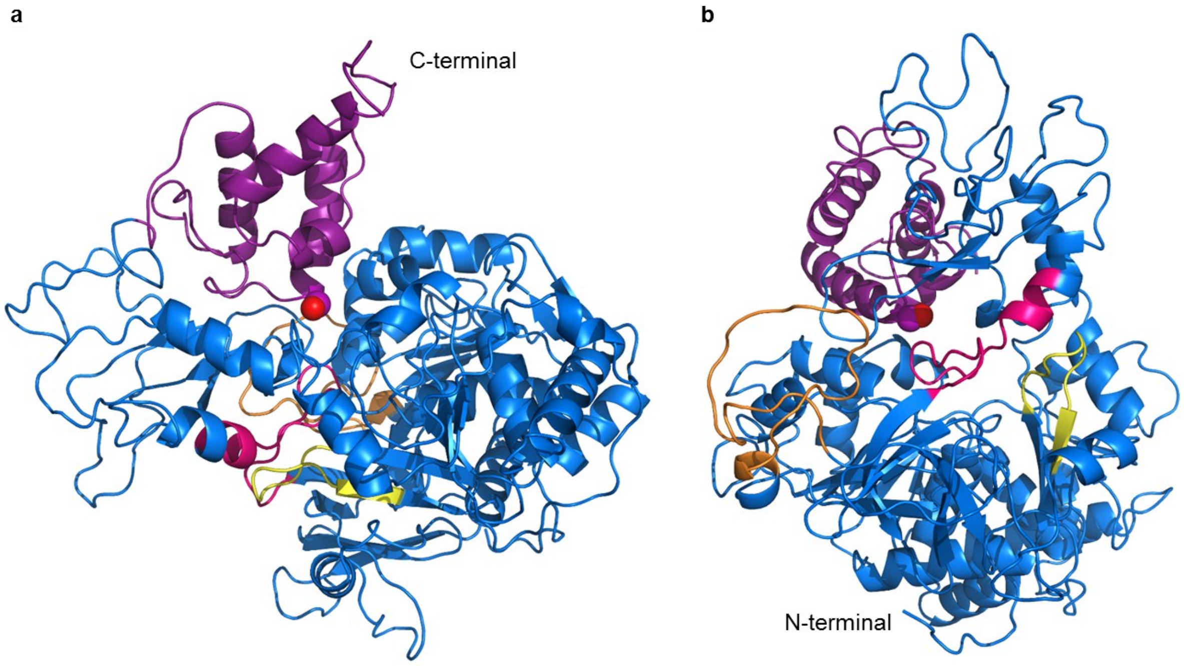

Figure S14: Model of the TamA structure showing the acyl carrier protein (ACP) domain in purple with the –OH of the 4′-phosphopantetheine (4′-PP) modified S622 residue shown as a red sphere, the adenylation domain (ANL) in blue with the hinge region (pink), the ATP-binding P-loop (yellow) and the fatty acid-AMP ligase (FAAL) insertion loop (orange). (**a**) Shows the S622 side chain at the top of helix 2 of the ACP sitting above the ANL central cavity and active site. A 4′-PP modification at this position would allow the 4′-PP arm to stretch into the active site. (**b**) An alternate view more clearly shows the ANL domain with the pink hinge region separating the large N-terminal and small C-terminal domains and allows rotation of the two with respect to one another. It also shows the yellow P-loop which is conserved in all ATP binding proteins and anchors ATP in the active site. Finally the FAAL insertion loop (orange) is modelled with very little confidence as it is not present in the structures used to build the model. However, it predicts a flexible loop that interacts with the ANL domain and stabilises the adenylation conformation. Binding of the cognate ACP domain results in conformational change and thiolation. This figure was drawn with Pymol Molecular Graphics System, Version 1.8.

Supplementary Table

Table S1: The Tam cluster open reading frames with GenBank codes, their homologues from the Red and Pig clusters for prodiginine biosynthesis[4,5], the predicted sizes of the resulting proteins and their conserved domains according to the BLAST conserved domain software.

**Gene**	**GenBank Code**	**Homologues**	**Predicted Size (kDa)**	**BLAST Conserved Domains**
tamA	EAR29369	-	75	Fatty acid AMP-Ligase/Acyl carrier protein
tamB	EAR29368	*pigG/redO*	10	Acyl carrier protein
tamC	EAR29367	redG	43	Rieske oxidase
tamD	EAR29366	*pigH/redN*	58	8-Amino-7-oxononanoate synthase
tamE	EAR29365	*pigI/redM*	56	Amino acid adenylation domain
tamF	EAR29364	*pigJ/redX*	81	Beta-ketoacyl synthase
tamG	EAR29363	*pigA/redW*	43	Acyl CoA dehydrogenase
tamH	EAR29362	*pigE*	104	Aminotransferase/Acyl-ACP reductase
tamI	EAR29359	-	50	ABC transporter permease
tamJ	EAR29361	*pigM/redV*	40	FMN nitroreductase
tamK	EAR29360	-	29	Periplasmic sorting protein
tamM	EAR29358	-	45	ABC transporter permease
tamN	EAR29357	-	26	ABC transporter ATPase
tamO	EAR29356	-	45	Unknown
tamP	EAR29355	*pigF/redI*	37	SAM-dependent methyltransferase
tamQ	EAR29354	*pigC/redH*	100	Phosphoenolpyruvate synthase
tamR	EAR29353	*pigK/redY*	12	Unknown
tamS	EAR29352	*pigL/redU*	25	Phosphopantetheinyl tranferase
tamT	EAR29351	-	62	Acyl-CoA dehydrogenase

Table S2: Deconvoluted masses for acylated TamA ACP from Figure 5 after reaction with TamA, fatty acid, Mg^{2+} and ATP. The table shows the observed and predicted masses of the proteins, as well as the observed and predicted mass changes expected for the addition of the acyl chain.

Fatty Acid Length	Observed Mass	Predicted Mass	Δ Mass	Δ Mass Predicted
Control	11306.2 +/-0.2	11307.8	/	/
C6	11404.4 +/-0.2	11405.9	98.2	98.1
C8	11432.6 +/-0.2	11433.9	126.4	126.1
C9	11446.5 +/-0.2	11447.9	140.3	140.1
C10	11460.5 +/-0.2	11461.9	154.3	154.1
C11	11474.7 +/-0.1	11476.0	168.5	168.2
C12	11488.7 +/-0.1	11490.0	182.5	182.2
C13	11502.3 +/-0.1	11504.0	196.1	196.2
C14	11515.9 +/-0.1	11518.0	209.7	210.2

References

(1) Liu, H.; Naismith, J. H. *Protein Expr. Purif.* **2009**, *63* (2), 102.

(2) Burke, C.; Thomas, T.; Egan, S.; Kjelleberg, S. *Environ. Microbiol.* **2007**, *9* (3), 814.

(3) Altschup, S. F.; Gish, W.; Miller, W.; Myers, E. W.; Lipman, D. J. *J. Mol. Biol.* **1990**, *215*, 403.

(4) Williamson, N. R.; Fineran, P. C.; Leeper, F. J.; Salmond, G. P. C. *Nat. Rev.* **2006**, *4* (12), 887.

(5) Hu, D. X.; Withall, D. M.; Challis, G. L.; Thomson, R. J. *Chem. Rev.* **2016**, *116*, 7818.
